# High SARS-CoV-2 Infection Rates Among Special Forces Police Units During the Early Phase of the COVID-19 Pandemic in Ecuador

**DOI:** 10.3389/fmed.2021.735821

**Published:** 2022-02-28

**Authors:** Esteban Ortiz-Prado, Felipe Andrade, Eduardo Vasconez, Cristina Escobar-Espinosa, Alexander Paolo Vallejo-Janeta, Byron Freire-Paspuel, Barbara Coronel, Heberson Galvis, Diana Morales-Jadan, Ismar A. Rivera-Olivero, Tannya Lozada, Aquiles R. Henriquez-Trujillo, Miguel Angel Garcia-Bereguiain, the UDLA-COVID-19 Team, Tatiana Jaramillo

**Affiliations:** ^1^One Health Research Group, Facultad de Ciencias de la Salud, Universidad de Las Américas, Quito, Ecuador; ^2^Dirección General de Investigación, Universidad de Las Américas, Quito, Ecuador

**Keywords:** SARS-CoV-2, RT-PCR, police, surveillance, Ecuador

## Abstract

**Background:**

At the beginning of the COVID-19 pandemic, health workers and first-responders, such as police officers, were in charge of trying to contain a disease that was unknown at that time. The lack of information and the tremendous need to contain new outbreaks put police officers at higher risk.

**Methodology:**

A cross-sectional study was conducted to describe SARS-CoV-2 infection rates among Police Special Forces Officers in Quito, Ecuador. In this study, 163 community-dwelling police officers from elite divisions voluntarily participated in our SARS-CoV-2 detection program using reverse transcription quantitative real-time PCR (RT-qPCR).

**Results:**

A total of 20 out of 163 police officers tested positive for SARS-CoV-2, yielding an infection rate of 12.3%. Within this cohort, 10% (2/20) of SARS-CoV-2 positive individuals were potentially super spreaders with viral loads over 10^8^ copies/ul. About 85% of the SARS-CoV-2 positive individuals were asymptomatic and 15% reported mild symptoms related to COVID-19.

**Conclusions:**

We found a high SARS-CoV-2 infection rate within the special forces police officers that, beyond a high health risk for themselves, their families, and coworkers. Our results point out the need for permanent SARS-CoV-2 testing among asymptomatic essential workers and first-responders to avoid local outbreaks and to prevent work-place absenteeism among police special units.

## Introduction

The COVID-19 pandemic caused by the new zoonotic coronavirus, the SARS-CoV-2 virus, has caused the worst health crisis worldwide, becoming one of the deadliest public health problems of this century (1). The rapid transmission, often driven by asymptomatic carriers, disseminated the virus globally. During the early phase of the pandemic, many low-and-middle-income countries (LMIC) faced the arrival of the virus before having the opportunity to prepare (2, 3). Poor epidemiological surveillance, limited testing capacities, and intensive care units (ICUs) working at full capacity were the norms (4).

In Latin America, the crisis was immense; fragmented health systems, weaker economies, and high demographic density as well as poverty, were the perfect formula for a disaster (5, 6). The earliest measure adopted by most countries was implementing country-wide lockdowns declaring the state of emergency (7). Implementing social distancing within communities required a multidisciplinary action plan, having police officers, special forces units, and the military deployed in every corner of the country. These security forces were permanently exposed to guarantee that control and prevention measures were followed by the population to reduce the spreading of COVID-19 (8, 9).

In spite of this, law enforcement agencies around the world are still facing unprecedented challenges due to the COVID-19 pandemic (10). Police officers, as security service providers, have been required to remain on duty during the entire length of the pandemic even during strict lockdowns worldwide (11, 12). Due to the nature of their activities, police officers are at higher risk of person-to-person spread through respiratory droplets and aerosols when close to someone coughing, sneezing, or talking (13). Among the police staff, special forces units included in our study may experience a higher occupational risk of exposure to COVID-19 as they cannot carry out many of their functions without being in close personal contact with others and may not have immediate access to all necessary personal protective equipment (PPE) and disinfection supplies in the field. Guidelines in accordance with the Center for Disease Control and Prevention (CDC) were placed among law enforcement worldwide. Nevertheless, 145 out of 264 police officers who died in the line of duty in 2020 were COVID-19 victims in the United States (14). In Latin America, dramatic numbers of SARS-CoV-2 infections and COVID-19 related deaths among police have been reported, with 534 victims in Peru, 465 victims in Brazil, and more than 1,000 soldiers and policemen infected in Colombia by June 2020 (15–17). In Ecuador, within the 1st month of the COVID-19 pandemic, there were 248 police officers and soldiers infected (3).

Under this scenario, the SARS-CoV-2 diagnostic laboratory at “Universidad de Las Américas” carried out a preventive screening among special forces police units in Quito (GOE/GIR), during July 2020, just a few weeks after the population lockdown was lifted. This study aimed to analyze the data obtained from this surveillance and describe the infection rates of SARS-CoV-2 infection among these essential workers to assess their potential occupational risk to COVID-19.

## Methods

### Study Design

A cross-sectional study was designed to describe SARS-CoV-2 infection rates among Special Forces Police Officers in Quito, Ecuador during July 2020. The participants were registered members of the police community, regardless of their symptoms, who decided to voluntarily participate in the SARS-CoV-2 screening program.

### Settings

**Figure 1:**
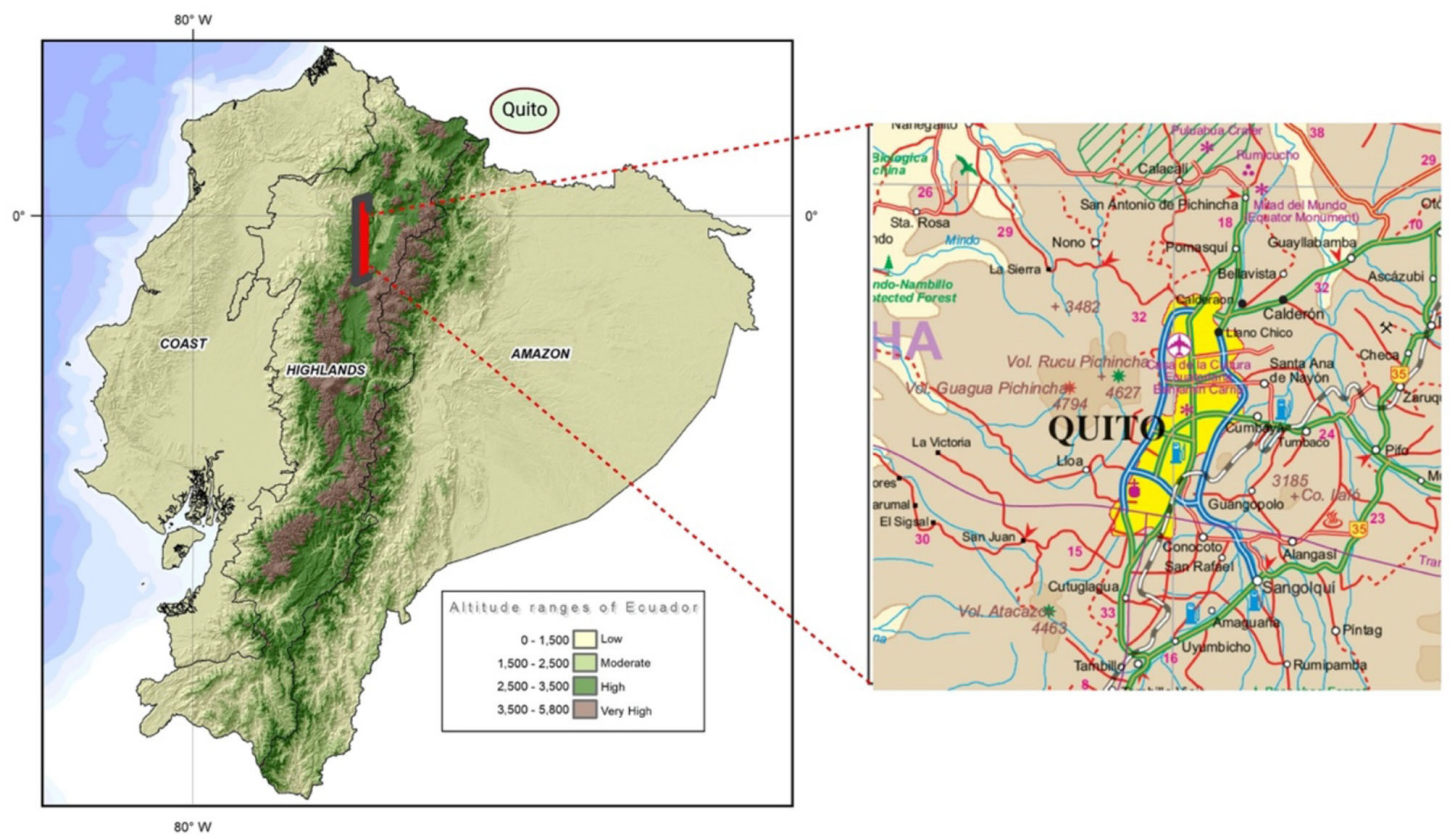
Map location of the population of study: 163 special forces police officers located in Quito, the capital city of Ecuador in the province of Pichincha (Highlands or Andean Region).

The study was carried out in Quito, the capital of Ecuador. The city is located at 2,850 m above sea level. According to 2020 projections, Quito has 2,781,641 million inhabitants representing 16% of the national population (as shown in Figure 1 for the map location of the study).

### Participants

A total of 163 members of the Intervention and Rescue Group (GIR) and Special Operation Group (GOE) divisions were recruited. Due to the highly specific roles of these groups, they are placed in special missions at high-risk situations, causing mobilization around the country to be unpredictable and with frequent turnover. So, this surveillance intervention was not designed for research aims.

### Study Size

We used a convenient non-probabilistic convenience sample including all the personnel available at the time. In the city of Quito, all police officers from GOE or GIR were formally invited to participate; however, some on duty did not attend the call. Sociodemographic information was obtained from the official epidemiological record used by the local health authority and the Ministry of Public health (MPH).

### Data Source and Variables

Using an informed consent as well as an epidemiological data recollection sheet form, demographic variables, such as sex, age, and symptomatology status were obtained.

**Figure 2:**
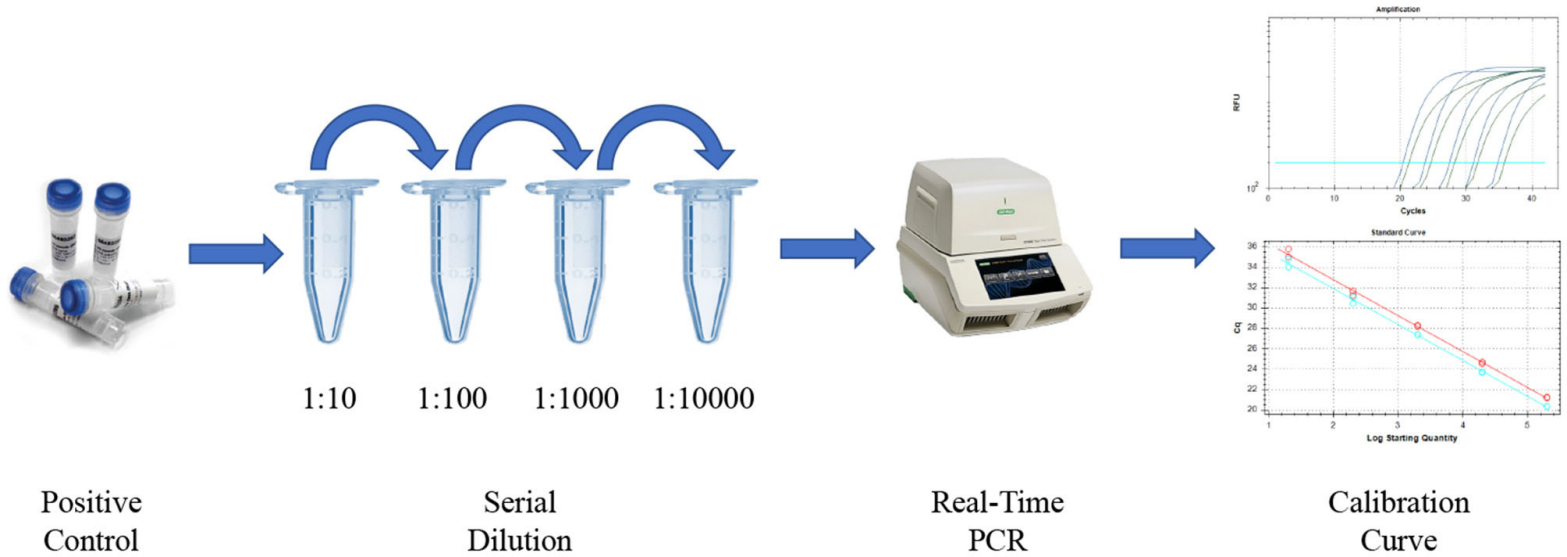
Diagram showing the protocol for viral load calculation in our study using 2019-nCoV N positive control (IDT, IA, USA). The reverse transcription quantitative real-time PCR (RT-qPCR) curves and regression plots are real ones for N1 and N2 viral targets for samples included in this study.

RNA extraction and reverse transcription quantitative real-time PCR (RT-qPCR) for SARS-CoV-2 detection using 2019-nCoV CDC kit. Nasopharyngeal swabs were collected on 0.5 ml TE pH 8 buffer for SARS-CoV-2 detection by reverse transcription quantitative real-time PCR (RT-qPCR) following an adapted version of the CDC protocol by using PureLink Viral RNA/DNA Mini Kit (Invitrogen, MA, USA) as an alternate RNA extraction method and CFX96 BioRad instrument (CA, USA) (18–25). Briefly, the CDC-designed RT-qPCR FDA EUA 2019-nCoV CDC kit (IDT, IA, USA) is based on N1 and N2 probes to detect SARS-CoV-2 and RNase P as an RNA extraction quality control (18, 19). In addition, negative controls (TE pH 8 buffer) were included as a control for carryover contamination, one for each set of RNA extractions, to guarantee that only true positives were reported. For viral loads calculation, the 2019-nCoV N positive control (IDT, IA, USA) was used, provided at 2,00,000 genome equivalents/ml. This positive control is a plasmid, such as N1 and N2 viral gene targets sequences, and it is a SARS-CoV-2 positive control recommended by CDC guidelines (18–25). As detailed in Figure 2, serial dilutions of the positive control were included in each set of samples RT-qPCR running, so an internal calibration curve with known concentrations of genomic SARS-CoV-2 material was always available. A regression analysis was made for each of those calibration curves taking RT-qPCR Ct values for N1 and N2 targets and viral genomic material concentrations as variables; the equation obtained was used for viral load calculations for each set of clinical, finally expressed as an average of the values for N1 and N2 targets. As it is exemplified in Figure 2, regression coefficients over 0.99 were obtained for the viral load calibration curves.

### Statistical Methods

Measurements of frequency (counts, absolute, and relative percentages), central tendency (median), dispersion [interquartile range (IQR)], and absolute differences were calculated for all categorical and continuous variables. Infection rates were compared using the chi-square test between age and sex groups. Finally, non-parametric tests were used to compare differences between viral loads across categories. The Kruskal–Wallis test was used to compare differences in median ages, Mann–Whitney *U*-test was used to compare medians between men and women. Statistical significance was accepted with *p* < 0.05. The analysis of the data was performed using the SPSS statistics software for Mac (IBM Corp. 2014, version 24.0. Armonk, NY, USA). Figures and graphs were performed in Prism 8 GraphPad Software version 8.2.0 (2365 Northside Dr. Suite 560, San Diego, CA, USA 92108). The basic cartography maps were generated using QGIS Development Team 2.8 (Creative Commons Attribution-ShareAlike 3.0 license CC BY-SA, CA, USA).

### Bias

To reduce the selection bias in this study, we have clearly described the specific targeted population (special police officers units deployed in Quito). On the other hand, to reduce the risk of any type of selection bias, we invited all the participants, regardless of their symptomatology status. Finally, we compared the results with other external populations to try to reduce the risk of information bias. To additionally reduce any type of bias, we used the standardized collection data sheet proposed by the national health ministry (Ministry of Health, MoH), the one that is applied to the entire population of Ecuador.

### Ethics Statement

All participants signed informed consent to participate freely and voluntarily in the molecular diagnosis of SARS-CoV-2. This study received the approval of the Ecuadorian MoH certified IRB.

## Results

**Figure 3:**
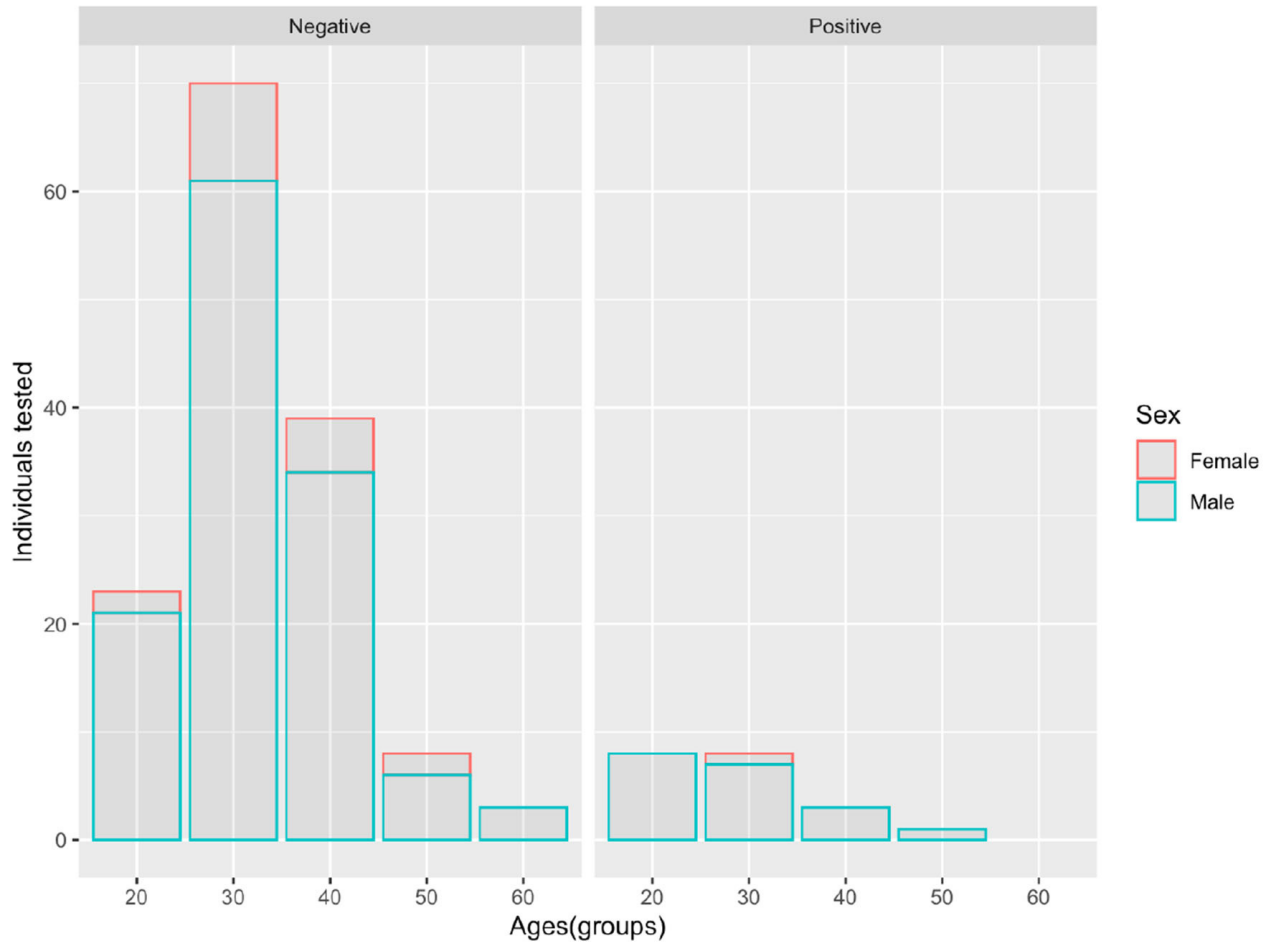
Age and sex distribution for SARS-CoV-2 RT-qPCR positive tests among the 163 special forces police officers included in the study (Viral load is expressed as log_10_ copies/ml).

In the city of Quito, we covered 81% of the total number of special forces officers. Within this cohort, 88.3% (144/163) of the police officers were men and 11.7% (19/163) were women police officers. The average age for the policemen and policewomen recruited was 37 and 39 years, respectively (Figure 3).

### Infection Rate

A total of 20 out of 163 police officers tested positive for SARS-CoV-2 RT-qPCR, yielding an infection rate of 12.3% (Table 1). The SARS-CoV-2 infection rate was significantly higher (*p* < 0.01) in men than in women, with values of 13.2 (19/144) and 5.3% (1/19), respectively. It was shown that 17 out of 20 (85%) of SARS-CoV-2 positive police officers were asymptomatic, while three of them (15%) reported mild symptoms related to COVID-19.

**Table 1:** SARS-CoV-2 reverse transcription quantitative real-time PCR (RT-qPCR) test results distribution among the special forces police unit officers included in the study.

			**SARS-CoV-2 RT-qPCR**		**Total**
			**Negative**	**Positive**	
Sex	Male	Count	125	19	144
		% within result	87.4%	95.0%	88.3%
	Female	Count	18	1	19
		% within result	12.6%	5.0%	11.7%
Total	Count	143	20	163
	% within result	100.0%	100.0%	100.0%

### Viral Load Analysis

**Figure 4:**
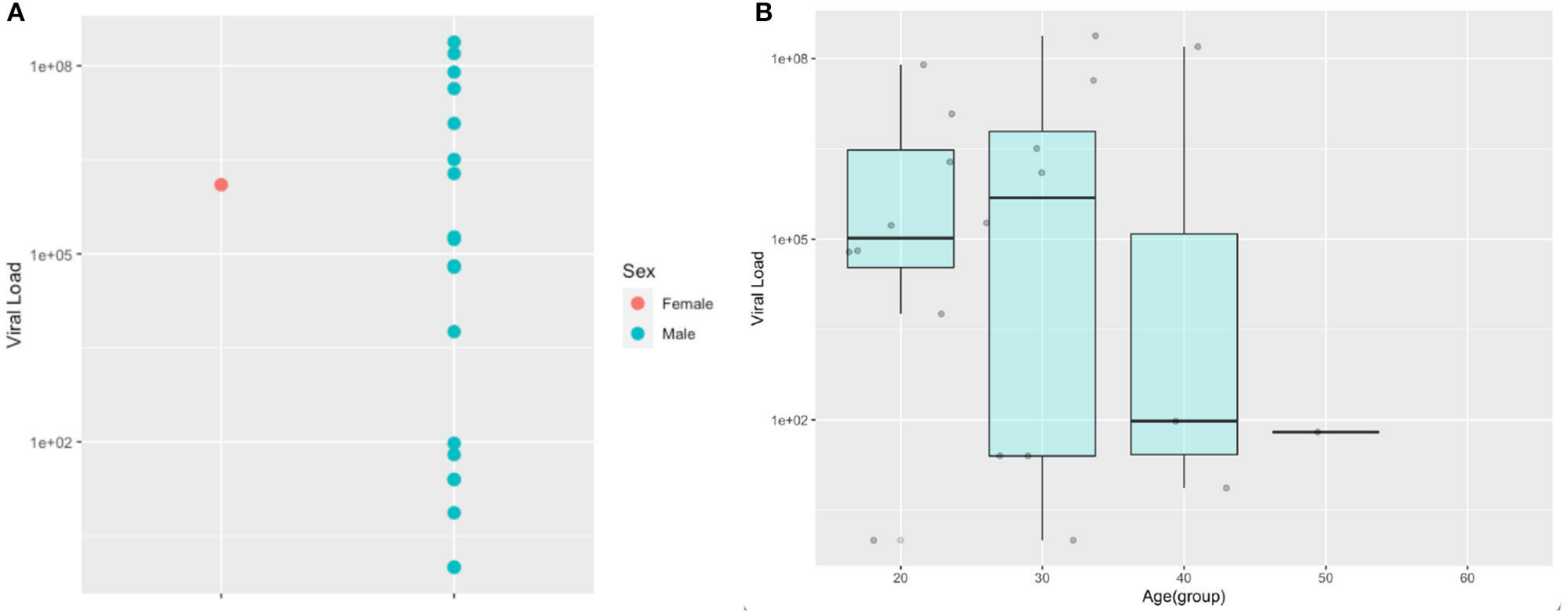
Viral load distribution for SARS-CoV-2 police officers according to sex **(A)** and age groups **(B)**.

The SARS-CoV-2 viral load's distribution for the positive individuals is detailed among sex and age groups. No significant differences were found for gender (*p* = 0.664) and age (*p* = 0.979). Additionally, there are two SARS-CoV-2 positive individuals with viral loads above 10^8^ copies/ml (Figure 4) that can be considered super spreaders.

## Discussion

Since the WHO declared the COVID-19 pandemic, many countries around the world have taken drastic measures, establishing public regulations to slow viral transmission. Several countries chose to declare a state of emergency, prior to the arrival of the virus to avoid a rapid explosion of contagion (1). Despite these measures, the virus spread rapidly in several places, prompting the government to implement more aggressive measures, such as the use of law enforcement to prevent population crowding (26, 27).

During the entire length of the pandemic, most countries deployed their police and police special units to ensure order and to prevent the spread of the disease in the population (14, 26). In March 2020, the government of Ecuador decided to declare a state of emergency establishing a population lockdown (3). However, after the lockdown restrictions were lifted, scenarios in which disorder, overcrowding, and non-compliance with social distance among the population prevailed in places, such as markets, public transportation, and population traveling from the cities to the countryside (3). This not only increased the risk of transmission among citizens but also puts police officers at risk, who in the performance of their work, try to enforce government regulations sometimes with inadequate precautionary measures and insufficient protection (28, 29).

While total COVID-19 cases and deaths worldwide have been regularly monitored and reported, less is known from the contribution to this number related to occupational risk for different types of workers. This is particularly worrying about essential workers, such as police officers or the military, as severe COVID-19 outbreaks among these groups may compromise their ability to work on the field. For instance, in our neighboring country of Peru, there were 1,300 positive cases and 11 deaths from COVID-19 among police officers by April 2020; those numbers rose to 4,098 cases and 82 deaths by May 2020; a dramatic number of 524 deaths of police officers was reported up to May 2021 (30). Another example comes from India, where up to August 2020, there were 71,832 infected and 428 deaths of police officers reported (31). Moreover, this is not an issue necessary associated with developing countries, as in the United States, more than 2,000 law enforcement officers had already been tested positive for SARS-CoV-2 by April 2020 (10). Our results confirm severe COVID-19 outbreaks among community dwelling special forces police officers with a high infection rate of 12.3% in Ecuador, suggesting that a massive COVID-19 transmission could be happening among other police officer groups or the military. Moreover, we found 2 individuals among the 20 SARS-CoV-2 positive ones, which means 10% of the infected population with viral loads above 10^8^ copies/ul that would represent community dwelling COVID-19 super spreaders (32–34).

Our results point out the need to improve SARS-CoV-2 testing among asymptomatic individuals at high occupational risk, such as the special forces police units included in this study. This group has a higher infection rate than healthcare workers in Ecuador, where the infection rate did not exceed 5% at the worst of the pandemic, according to official data from the Ecuadorian MoH. On the other hand, is a highly mobile group, with scarce economic resources and limited access to healthcare, such as delivery food-riders, we found very similar levels of 15% SARS-CoV-2 infection rate (35). In the only study available in Ecuador regarding occupational risk from official data by the Ministry of Health, our research group found that police and military personnel were almost two times more prone to die from COVID-19 than the general population within the same age range (3).

Under this scenario, additional measures to prevent the spreading of COVID-19 among police officers must be reinforced, such as sanitization of equipment used at work, cleaning of clothes and personal items before returning home, and use of proper disposable PPE, as recommended by the US CDC or INTERPOL. The Ecuadorian National Police has included these recommendations in its own guideline, nevertheless, police officers are always at risk of not being able to use them properly. Additionally, informational resources, such as the ones offered in the United States from the National Police Foundation, could provide updated regionalized information and all types of support for officers that are constantly exposed and at increased stress regarding their safety (National Police Foundation). However, law enforcement officers cannot carry out many of their duties without being in close personal contact with others and may not have immediate access to all necessary disinfection supplies in the field.

As the high prevalence of SARS-CoV-2 infection among police officers found in our study suggests, the precautionary measure may not be enough to protect police officers against COVID-19, and regular SARS-CoV-2 testing should be mandatory as they are constantly enduring public measures. Moreover, our results endorsed including police officers among priority groups for SARS-CoV-2 vaccination. By May 2021, more than 1,00,000 police officers and military personnel received the first dose of the SARS-CoV-2 vaccine in Ecuador (36). Those protective measures against COVID-19 for police officers and the military, particularly for special forces units described in this study, should be considered a matter of national security. For instance, police officers have been answering thousands of emergency calls related to public order disturbance and crowd control during the COVID-19 pandemic, and they had to act fast to restore order and implement social distancing measures (30). In Ecuador, the initial reduction in crime rates during the population lockdown was followed by an exponential increase in crime that demanded security forces at full capacity (37).

### Limitations

The main limitation of this study is that we lack timely and accurate information on PPE used among police officers. The information related to PPE could be linked to the high rates of infection due to the lack of efficient protection while on duty. Also, another important limitation is that viral load was not analyzed in relation to the day of symptom onset, therefore, it is not possible to calculate correctly when the peak of transmission was reached in one or another person. Finally, the use of convenience sampling means that there is a risk for selection bias. However, due to the high response rate, the impact of this bias was probably limited.

## Conclusion

Our study supports that police officers are at high risk of occupational exposure to COVID-19, so they should be regularly tested for SARS-CoV-2 infection and included as a priority group for vaccination, not only to protect themselves, their families, and the community, but also as a matter of national security under the unfortunate scenario of social crisis imposed by COVID-19 pandemic.

## Data Availability Statement

The original contributions presented in the study are included in the article/supplementary material, further inquiries can be directed to the corresponding author/s.

## Ethics Statement

This study was reviewed and approved by the Ecuadorian MoHcertified IRB, code: CEISH-HGSF-2021-002. All participants signed written informed consent to participate freely and voluntarily in the molecular diagnosis of SARS-CoV-2.

## UDLA COVID-19 Team

Tatiana Jaramillo, Daniela Santander Gordon, Gabriel Alfredo Iturralde, Julio Alejandro Teran, Karen Marcela Vasquez, Jonathan Dario Rondal, Genoveva Granda, Ana Cecilia Santamaria, Cynthia Lorena Pino, Oscar Lenin Espinosa, Angie Buitron, David Sanchez Grisales, Karina Beatriz Jimenez, Vanessa Bastidas, Dayana Marcela Aguilar, Ines Maria Paredes, Christian David Bilvao, Maria Belen Paredes-Espinosa, Angel S. Rodriguez, Juan Carlos Laglaguano, Henry Herrera, Pablo Marcelo Espinosa, Edison Andres Galarraga, Marlon Steven Zambrano-Mila, Ana Maria Tito, Nelson David Zapata.

## Author Contributions

EO-P and MG-B were fully responsible for the conceptualization of this investigation. AH-T, IR-O, TL, and AV-J contributed to the process of data collection and raw data processing. FA, CE-E, and EV were responsible for part of the data analysis and graphical display of the manuscript. EO-P and FA were responsible for the construction of some figures and tables and they contributed equally with the descriptive statistical analysis. EO-P, EV, and MG-B elaborated the first draft of the manuscript. TL, AH-T, and IR-O added an important insight on the overall manuscript. All authors critically reviewed the entire final version of the manuscript, produced several comments prior to the submission, and participate in data collection and primary analysis.

## Funding

This study was funded by Universidad de Las Américas and by Fundación CRISFE (Fondo Sumar juntos).

## Conflict of Interest

The authors declare that the research was conducted in the absence of any commercial or financial relationships that could be construed as a potential conflict of interest.

## Publisher's Note

All claims expressed in this article are solely those of the authors and do not necessarily represent those of their affiliated organizations, or those of the publisher, the editors and the reviewers. Any product that may be evaluated in this article, or claim that may be made by its manufacturer, is not guaranteed or endorsed by the publisher.
